# First report of *Kudoa* species (Myxozoa, Multivalvulida) infection in purple-spotted Bigeye (*Priacanthus tayenus*) from the Saudi Arabian Gulf

**DOI:** 10.1371/journal.pone.0295668

**Published:** 2024-01-10

**Authors:** Mustafa M. Ibrahim, Marwa M. Attia, Hanadi B. Baghdadi, Mohamed Abdelsalam

**Affiliations:** 1 Department of Pathology, Animal Health Research Institute, Dokki, Giza, Egypt; 2 Faculty of Veterinary Medicine, Department of Parasitology, Cairo University, Giza, Egypt; 3 Faculty of Science, Department of Biology, Imam Abdul Rahman bin Faisal University, Dammam, Saudi Arabia; 4 Faculty of Veterinary Medicine, Department of Aquatic Animal Medicine and Management, Cairo University, Giza, Egypt; Sher-e-Kashmir University of Agricultural Sciences and Technology of Kashmir, INDIA

## Abstract

The purple-spotted bigeye, *Priacanthus tayenus*, is a marine benthic fish native to the Indian and Pacific Oceans, including the Arabian Gulf in Saudi Arabia. This study identified a myxozoan parasite infecting wild *P*. *tayenus* from the Saudi Arabian Gulf. These parasites produced spherical to ovoid-shaped, white plasmodia enclosed within pseudocysts in the fish musculature. The annual infection rate was 5.1%, with the highest prevalence in summer (7.6%), followed by spring (6%), and autumn (2.5%), while no infections were observed in winter. The number of plasmodia per fish ranged from 100 to 150 (135.1 ± 16.2). Their dimensions were 4–4.7 mm (4.3 ± 0.3 mm) in length and 4.5–7 mm (6 ± 1.1 mm) in width. Milky-colored exudates within the plasmodia contained mature spores measuring 8–9 μm (8.6 ± 0.4 μm) x 6–7.5 μm (6.9 ± 0.5 μm). The polar capsules of the spores exhibited dimensions of 2–5 μm (3.5 ± 0.5 μm) x 2.5–4.5 μm (3 ± 0.45 μm). Both morphological and genetic analyses confirmed these plasmodia as a novel *Kudoa* species. Histopathological examination revealed atrophy in the surrounding muscles without an inflammatory response. This study documents the first occurrence of a novel *Kudoa* sp. in *P*. *tayenus* at the Jubail landing site in Saudi Arabia, emphasizing the need for further surveillance and investigations to elucidate its pathogenesis and implications for wild fish stocks.

## 1. Introduction

The Arabian Gulf, a semi-enclosed sea bordered by eight countries in the Middle East, is among the most severely polluted marine ecosystems globally **[1].** Anthropogenic activities, elevated salinity, water evaporation, and temperature fluctuations pose significant challenges to the marine biodiversity of this region **[2].** Fish parasites are an essential part of this biodiversity **[3]** and can harm marine fish populations, affecting the quality and value of marine products **[4,5].**

Myxosporeans (Cnidaria, Myxozoa) are destructive parasites that affect marine fish musculature, causing fish to be rejected from commercial markets **[6].** These parasites can also impact various fish organs **([7,8]**. Within the Myxosporean group, the monotypic Kudoidae family (Myxozoa: Myxosporea) comprises the single genus, *Kudoa*. *Kudoa* species are globally distributed marine endoparasites infecting various fish species **[9].** These species are characterized by four or more shell valves forming a thin membrane in a quadrate or stellate pattern. Some documented *Kudoa* spp. have up to 13 shell valves, each containing a polar capsule visible under a microscope **[6].** *Kudoa* spp. were later recognized as a distinct genus characterized by unique features, including a pair of sporoplasm cells, one encasing the other **[10].**

Some *Kudoa* spp. exhibit site-specificity when entering the integument of the host, either establishing themselves in body cavities (coelozoic) or within tissues (histozoic). Coelozoic species generally do not prompt significant host responses, while histozoic species are more often associated with pathological reactions. Pseudocyst-forming species can induce similar host reactions **[11]**. Severe cases of *Kudoa* spp. infections are a global concern in the fishing sector, often linked to myoliquefaction and the development of multiple plasmodia in fish muscles **[12]**. Notably, *Kudoa thyrsites*, a widely distributed myxosporean species, is known to induce "soft flesh" in affected fish **[13]**, leading to reduced market value and significant economic losses **[14].**

*Priacanthus tayenus*, the purple-spotted bigeye, is a commercially important marine ray-finned fish found in the Pacific and Indian Oceans and the Arabian Gulf. Despite its commercial value, *P*. *tayenus* has not been extensively studied regarding its parasitological aspects in the Arabian Gulf. Previous studies in Saudi Arabia mainly focused on Kudoidae in marine fish, with no records of their presence in purple-spotted bigeye specimens from the Jubail landing site in the Eastern part of the Saudi Arabian Gulf **[13,15]**. Therefore, investigating the parasitological aspects of *P*. *tayenus* and their histopathological effects on the fish is crucial, especially when *Kudoa* spp. infections are identified in commercially important fish species.

This study revealed the presence of multiple plasmodia of myxosporea in the musculature of purple-spotted bigeye, significantly impacting the quality of affected fish and market value. Our study aimed to provide a detailed morphological and molecular characterization of this myxosporean parasite, offering insights into the histopathological responses of the host to this myxosporean infection.

## 2. Materials and methods

### Ethical approval

Ethical Approval: Approval for this study was granted by the institutional animal care and use committee (IACUC) of Cairo University, Faculty of Veterinary Medicine (Vet CU 25122023870). The collection and analysis adhered to guidelines from the Veterinary Office within the Welfare of Fisheries division in Jubail Province, Saudi Arabia.

### Fish sampling

Between January 2018 and December 2019, we collected 175 specimens of *P*. *tayenus* in the coastal waters of the Arabian Gulf, specifically in Jubail Province, Saudi Arabia (coordinates: 27° 02′ 20.5′′ N 49° 38′ 18.9′′ E). The first author, an authorized representative from the Veterinarian Office within the Welfare of Fisheries division at Jubail province, Ministry of Agriculture, conducted routine fish inspections from landing stations to fish markets and shops in Jubail. Infected fish were isolated in plastic bags with ice for preservation during transportation to the Jubail Welfare Fish branch laboratory. These fish, averaging 20–25 cm in length and 300–400 g in weight, were filleted. Moribund fish with multiple white plasmodia in their skeletal muscles were selected, and samples of infected tissues were stored at -80°C for molecular analysis.

### Morphological examination

The morphological analysis of *Kudoa* sp. spores was conducted following the methods described by **[16,17]**. Wet mount slides were prepared by compressing the white plasmodia between two slides and examined under a light microscope at a magnification of 400X. Additionally, the parasite plasmodia underwent Giemsa staining after air-drying **[18,19]**. The morphological analysis and measurement of 20 *Kudoa* sp. spores used multiple digital images captured with an Olympus BX50 light microscope within a magnification range of 400X to 1000X **[20].**

### PCR amplification of 18S rRNA

For genetic characterization, we extracted genomic DNA from 40 mg of plasmodia collected from infected fish at the same locality over a year. DNA extraction utilized the DNeasy® Blood and Tissue Kit (Qiagen) following the manufacturer’s protocol. The concentration and purity of the extracted DNA were evaluated with a NanoDrop2000 spectrophotometer (Thermo Fisher Scientific), and the DNA was stored at -20°C. PCR amplification of the 3′ half of the 18S rDNA gene was accomplished with the Myxospec F: 5′-TTCTGCCCTATCAACTWGTTG-3′ **[21]** and universal 18R: 5′-CTACGGAAACCTTGTTACG-3′ **[22]** primer pair, which showed the highest efficiency among several primer sets evaluated. PCR was performed using the MyTaqTM Red Mix kit (Bioline) in a 25 μL reaction, including 0.2 μg of DNA template, 10 pmol of each primer, and nuclease-free water. The PCR cycling program consisted of an initial denaturation at 95°C for 3 minutes, followed by 35 cycles of 94°C for 30 seconds, 55°C for 30 seconds, and 72°C for 2 minutes, with a final extension step at 72°C for 10 minutes **[23].** The amplicons were then separated by electrophoresis on a 1% agarose gel, excised with a sterile scalpel, and purified using the QIAquick Gel Extraction Kit (QIAGEN).

### Partial sequencing of 18S rDNA

Purified PCR products underwent bi-directional sequencing with the Big Dye® Terminator v3.1 kit (AB Applied Biosystems) using the same PCR primers. Sequences were capillary electrophoresed on ABI PRISM3130 automated sequencers (Applied Biosystems, USA). Raw sequences were edited and assembled in Unipro UGENE version 33 **[24].** Assembled contigs were aligned against GenBank 18S rDNA sequences via BLASTN. The resulting nucleotide sequences were deposited in GenBank and assigned accession numbers.

### Phylogenetic analysis

In phylogenetic analysis, we compared current 18S rDNA partial sequences to 29 accession numbers from various *Kudoa* species, each exhibiting over 85% similarity. To root the tree, *Ceratomyxa shasta* was used as an outgroup. Multiple sequence alignment employed the Clustal W program. Phylogenetic relationships were established through both maximum likelihood (ML) and Bayesian inference (BI) methodologies. For the ML analyses, we utilized MEGA 11 **[25].** ML parameters were determined using the general time reversible model with gamma-distributed rate and invariant sites (GTR + G + I model). Model selection relied on Bayesian information criterion (BIC), corrected Akaike information criterion (AIC) scores, and bootstrap confidence values from 1,000 replicates.

Bayesian analyses were conducted with MrBayes v3.2.6 **[26]**. BI analyses employed the general time reversible model with gamma-distributed rate and invariant sites (GTR + G + I). Parameters included 1,000,000 generations with tree sampling every 100th generation. The initial 25% of trees were discarded as the ’burn-in’ phase, and the remaining data were used to construct consensus trees with the 50% majority rule and posterior probabilities (PPs).

### Histopathological analysis

Histopathological analysis involved carefully trimming skeletal muscle samples obtained from infected *P*. *tayenus*. These samples were preserved in a 10% neutral buffered formalin solution, embedded in paraffin, sectioned into four-micrometer-thick sections, stained with hematoxylin and eosin (H&E), and examined using a compound light microscope (Olympus BX50, Japan) **[27]**.

### Inclusivity in global research

The questionnaire file contains additional information concerning the ethical, cultural, and scientific considerations that are specific to promoting inclusivity in global research.

## 3. Results

**Host:** *Priacanthus tayenus*, (purple-spotted bigeye).

**Locality:** The Jubail landing site on the Eastern part of the Saudi Arabian Gulf.

**Site of infection:** Skeletal muscles.

**Incidence:** (9/175) accounting for a 5.1% incidence.

**DNA sequences:** OQ170970; OR242261; OR242262 and OR242263.

### Parasite investigation

**Fig 1:**
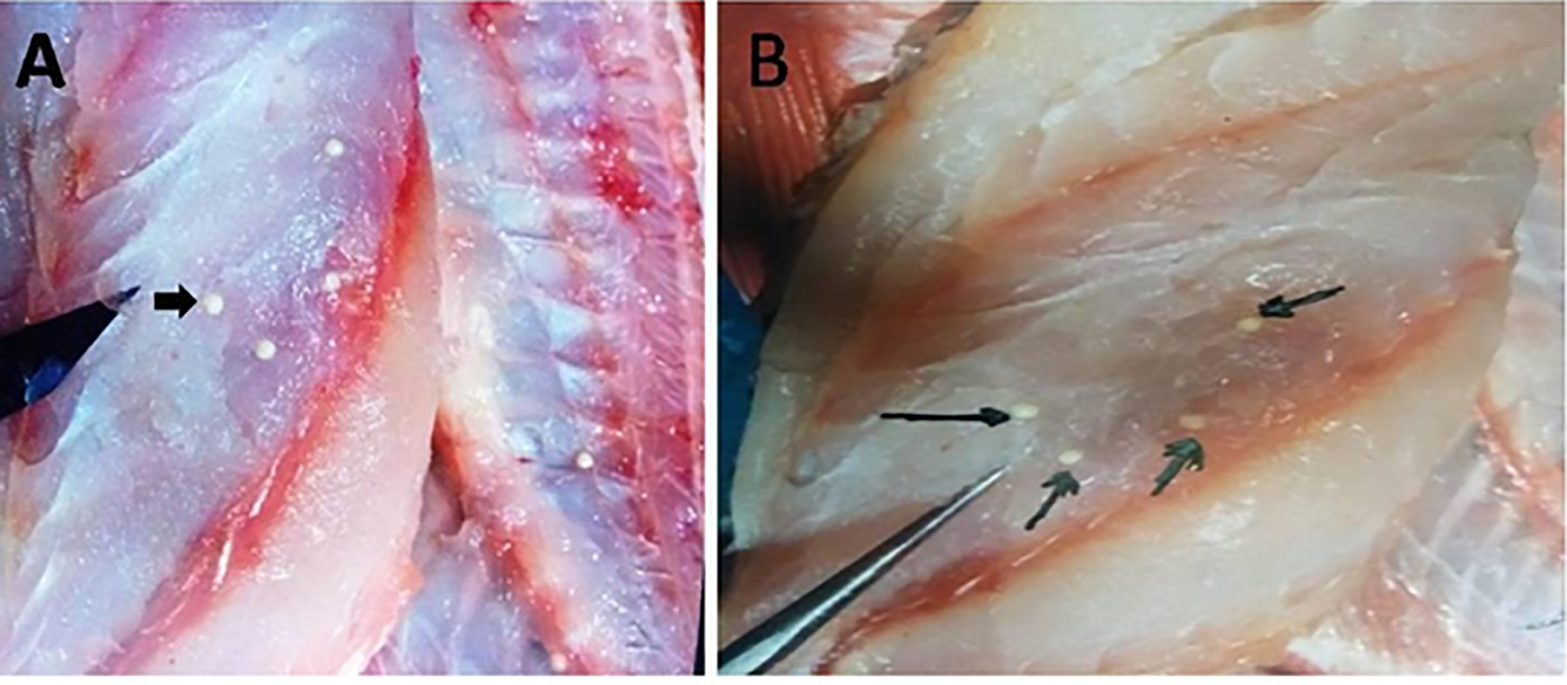
Macroscopic examination of heavily infected skeletal muscle in *P*. *tayenus*, revealing the presence of white plasmodia (*Kudoa* plasmodia).

**Fig 2:**
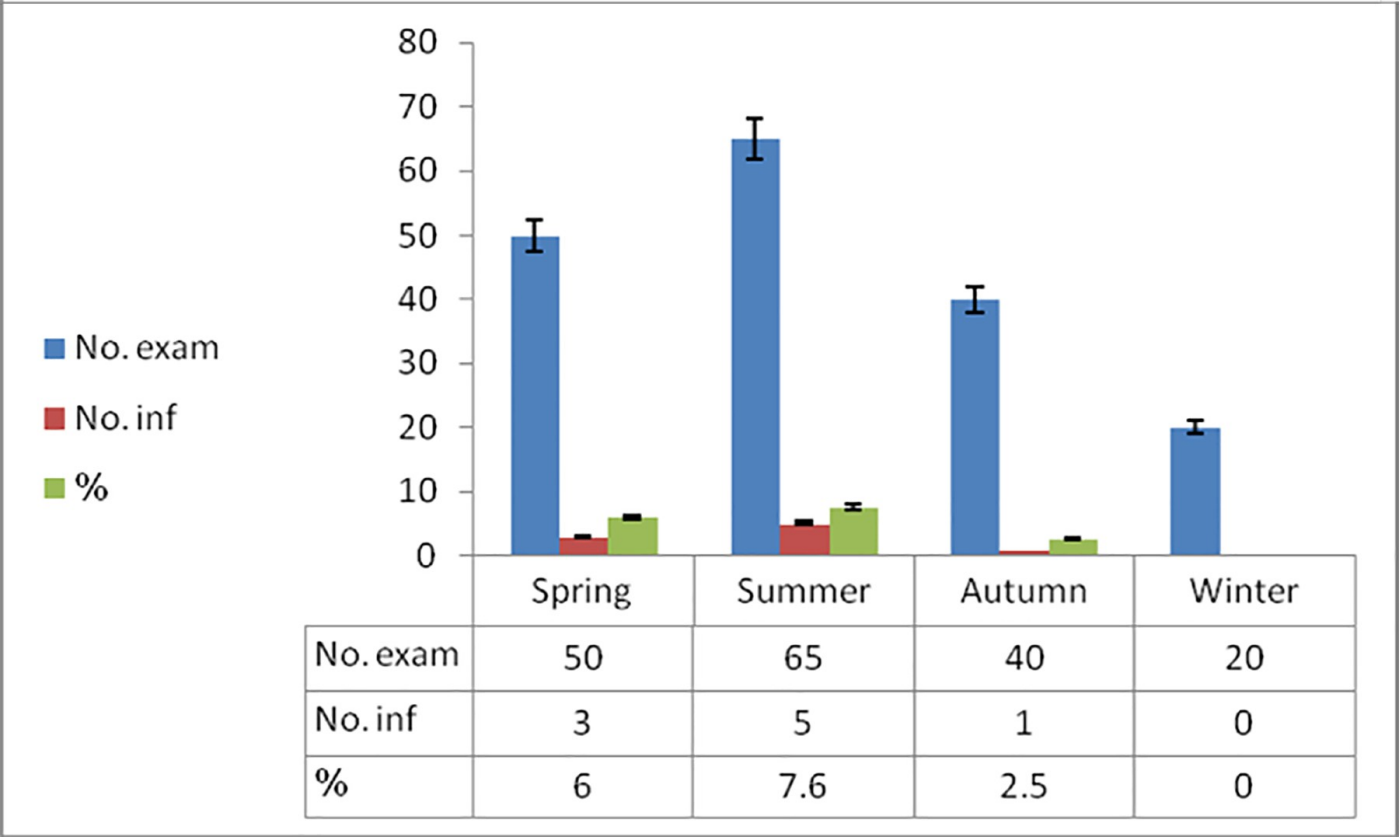
Seasonal variation in the frequency distribution of *Kudoa* sp. infection within skeletal muscles of *P*. *tayenus* (Total specimens examined: 175; total positives: 9; overall prevalence: 5.1%).

**Fig 3:**
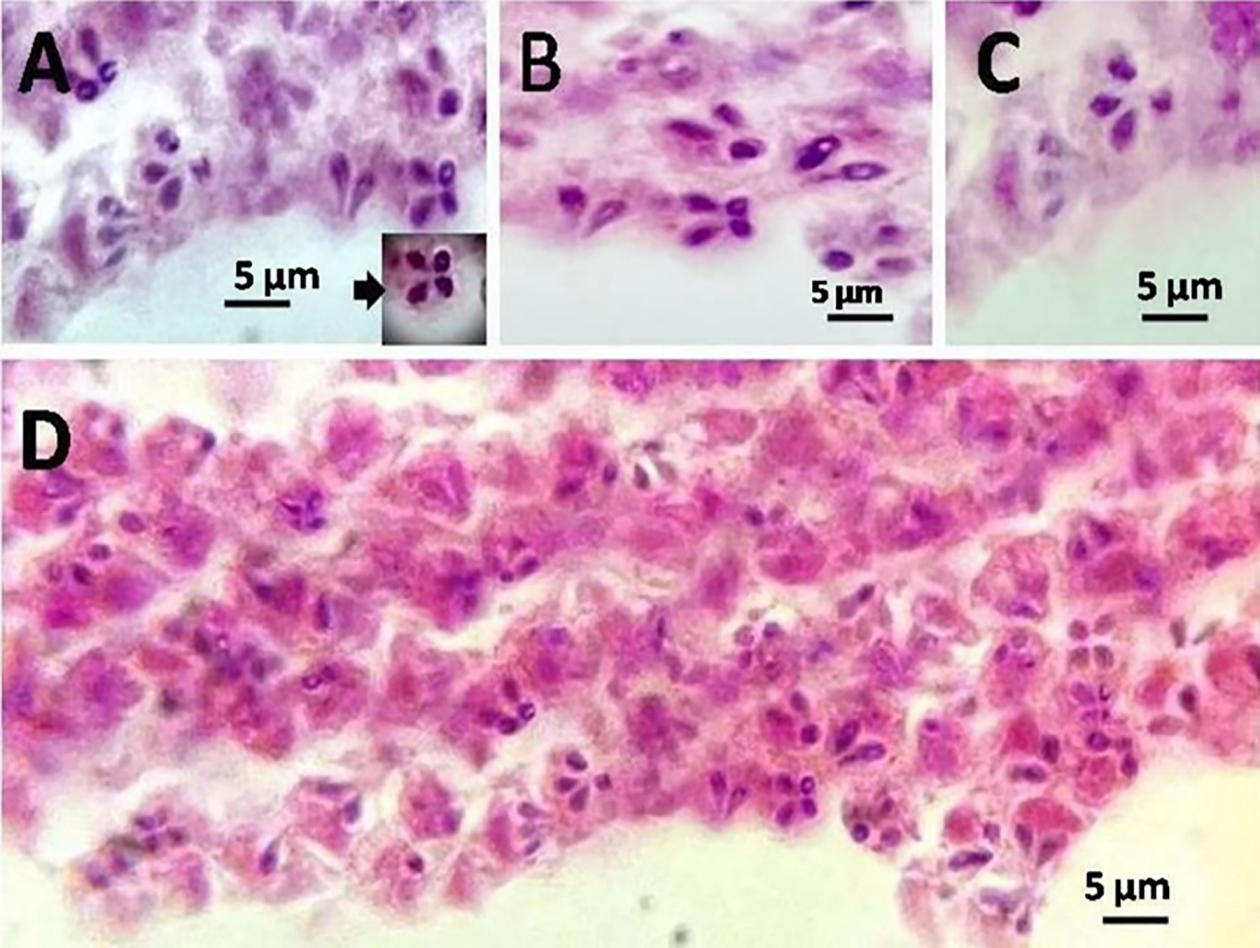
*Kudoa* sp. within the muscles of *P*. *tayenus*; displaying mature *Kudoa* spores with a quadrangular shape and rounded ends. Panels A-D illustrate the four thin, smooth valves forming the spore wall, enclosed by four pyriform polar capsules of equal size.

During the necropsy and filleting process of the examined moribund *P*. *tayenus* specimen, white, rounded, oval multiple plasmodia were observed within the myofibers of the skeletal muscle (**Fig 1**). These plasmodia displayed irregular sizes and were randomly distributed, with no infection observed in internal organs. Among the 175 samples examined, nine were infected, accounting for a 5.1% incidence. Seasonal variation in infection prevalence was noted, with rates of 7.6% in summer, 6% in spring, and 2.5% in autumn (**Fig 2**). No infections were reported during the winter season. The number of plasmodia per fish ranged from 100 to 150 (135.1 ± 16.2), indicating a high intensity of plasmodia per fish. These plasmodia exhibited variable dimensions, with lengths ranging from 4–4.7 mm (4.3 ± 0.3 mm) and widths from 4.5–7 mm (6 ± 1.1 mm). Wet compressed preparations of these plasmodia revealed mature spores characteristic of *Kudoa* sp. parasites, with four refractile polar capsules, and exhibiting positive staining when subjected to Giemsa stain **(Fig 3).** Milky-colored exudates within the plasmodia contained mature spores. These mature spores measured 8–9 μm (8.6 ± 0.4 μm) x 6–7.5 μm (6.9 ± 0.5 μm). The polar capsules of the spores exhibited dimensions of 2–5 μm (3.5 ± 0.5 μm) x 2.5–4.5 μm (3 ± 0.45 μm).

### Histopathological findings

**Fig 4:**
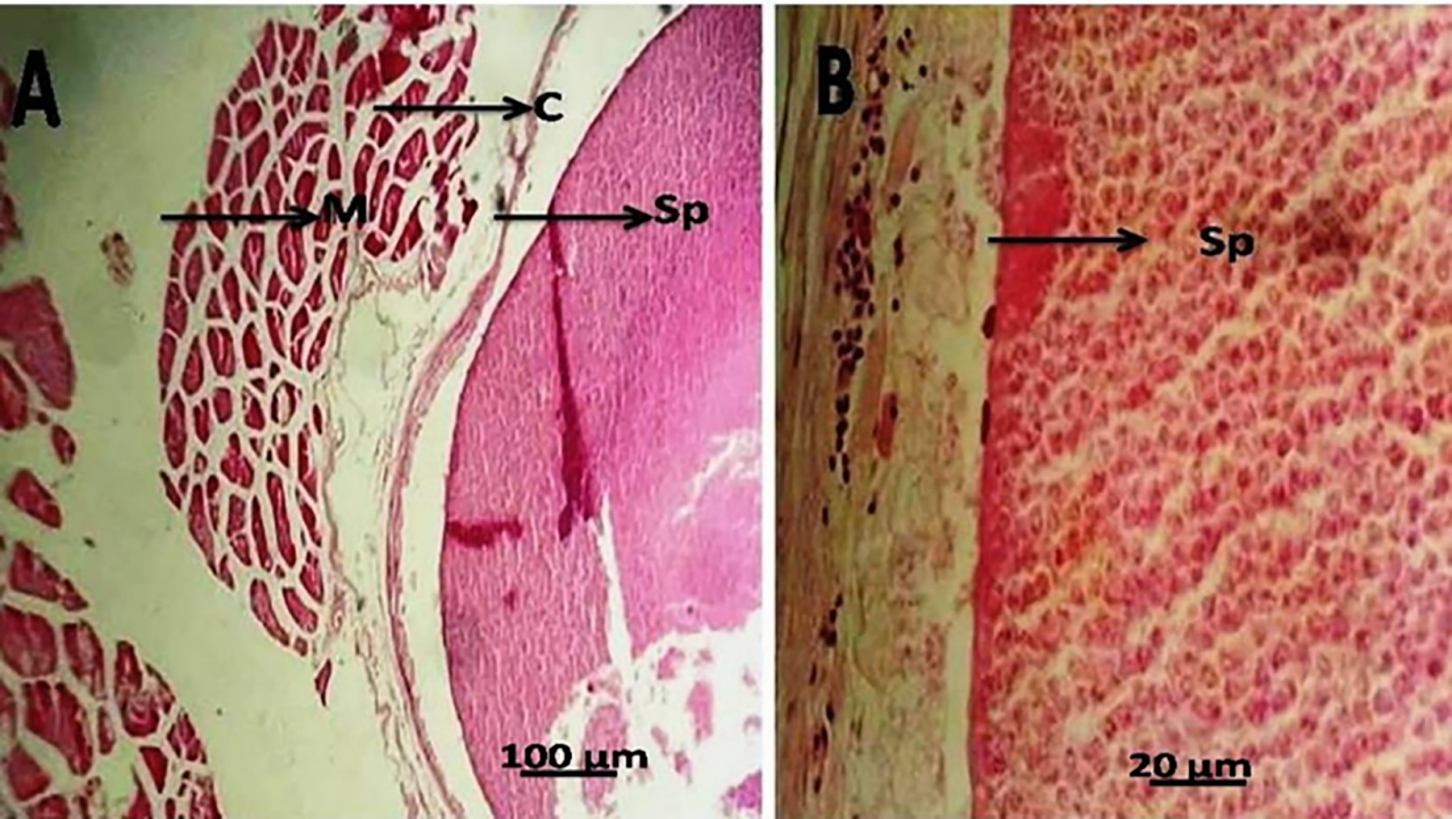
(A & B) Histopathological alterations in the skeletal muscle of *P*. *tayenus* fish (M) displaying connective tissue capsules filled with mature *Kudoa* spores (Sp) and associated muscular atrophy. Staining was performed using hematoxylin and eosin (H&E).

The histopathological analysis of the skeletal muscles affected by the white plasmodia demonstrated the presence of a dense connective tissue capsule surrounding the myxosporean spores. The surrounding muscles exhibited atrophy without any accompanying inflammatory response (**Fig 4**). The myofibers of the dorsal and abdominal muscles exhibited polysporic cysts characterized by their random distribution and irregular shape (**Fig 4**).

### Sequencing and phylogenetic tree

Amplification and partial sequencing of the 18S rDNA region from the myxosporean parasite infecting four different *P*. *tayenus* specimens resulted in a DNA fragment measuring 1391 base pairs (bp). These four sequences of the 18S rDNA region from the four fish were identical and deposited in the GenBank database under unique accession numbers (OQ170970, OR242261, OR242262, and OR242263). The myxosporean parasite isolated in this study was classified within the genus *Kudoa* (Myxozoa: Multivalvulida) as confirmed by sequence alignment analysis. The sequence (OQ170970) exhibited distinctions from other myxosporean 18S rDNA sequences.

The genetic analysis of the novel *Kudoa* sp. (OQ170970) demonstrated notable similarities with various *Kudoa* spp., signifying its taxonomic affiliation. Specifically, it exhibited a substantial similarity of 98.99% to *K*. *Alliaria* and 98.85% to *K*. *rosenbuschi*. Moreover, when compared to a range of *Kudoa* spp. strains (EU041621; EU041613; EU041614; EU041616; EU041617 and EU041620) known to infect muscular tissues of diverse fish species, the genetic sequence (OQ170970) displayed a similarity range of 98.99% to 98.91%. However, lower degrees of similarity were observed when compared to other *Kudoa* spp.: 96.99% to 95.97% for *K*. *thalassomi* isolates (HM022116 and AB844443), 96.64% to 96.33% for *K*. *thyrsites* isolates (AY382607; MT913637; AY542481 and MH899080), 95.06% to 94.93% for *K*. *iwatai* isolates (AY514039 and AB553294), and 94.68% for *K*. *musculoliquefaciens* isolates (LC097082 and LC097083).

**Fig 5:**
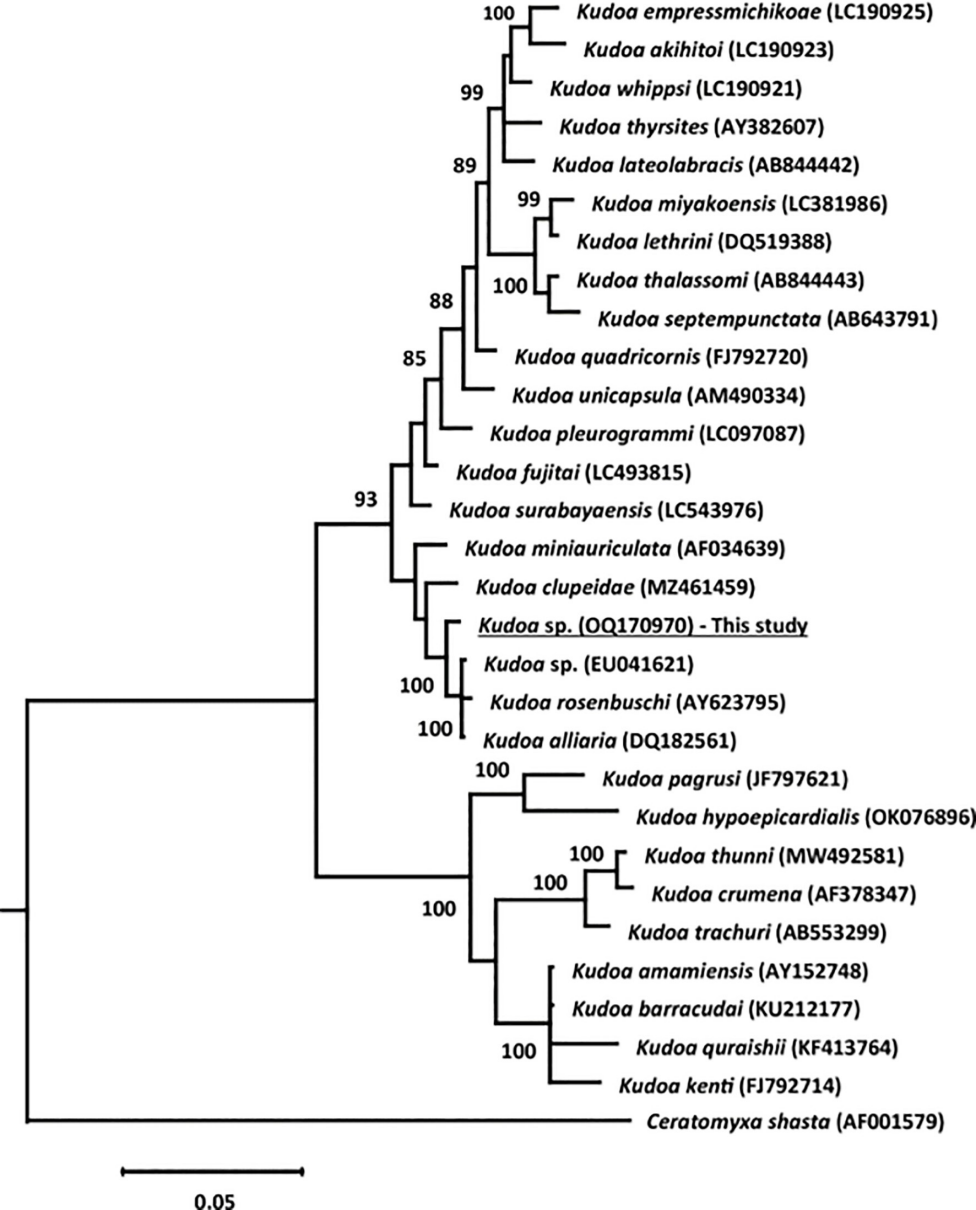
Phylogenetic tree obtained through Bayesian analysis for comparative analysis of the 18S rDNA gene sequence of *Kudoa* sp. in *P*. *tayenus* muscle fibers and related myxosporeans. Bayesian posterior probability support values are displayed as node values, with values exceeding 85%. The scale bar represents site/substitutions.

Phylogenetic trees were generated using Bayesian inference and maximum likelihood analysis, and both methods produced comparable topologies. The novel *Kudoa* sp. investigated in this study was taxonomically assigned within the same clade as other muscular-infecting *Kudoa* parasites, including *K*. *alliaria*, *K*. *rosenbuschi*, *K*. *clupeidae*, and *K*. *miniauriculata*. This assignment was supported by strong bootstrap values obtained through both applied techniques **(Fig 5).**

## 4. Discussion

In light of the increasing scientific research on parasites in Arabian Gulf fish populations, this study underlines the significant economic and health concerns associated with these parasites **[20,28].** *Kudoa* parasites primarily target fish skeletal muscles, potentially causing postmortem myoliquefaction or visible plasmodia on fillets, thereby reducing the quality of the fish products **[29].** Despite its economic importance, our knowledge of *Kudoa* biology and life cycles in aquatic environments remains limited **[4,30].**

In this study, distinctive white, multi-nucleated plasmodia (pseudocysts) belonging to the *Kudoa* genus were discovered in the skeletal muscles of *P*. *tayenus*. The number of these plasmodia varied, ranging from 100 to 150, with an average of 135.1 ± 16.2, reflecting varied density within the fish muscles, as observed in previous studies **[31].** Examination of compression smears from these plasmodia revealed spores with a rectangular quadrate morphology, similar to a star with four shell valves and four polar capsules, consistent with earlier findings **[32].**

The newly discovered *Kudoa* sp. exhibited plasmodial dimensions distinct from those of closely related species like *K*. *rosenbuschi* and *K*. *alliaria* found in Argentina. Specifically, *K*. *alliaria* displayed substantially larger plasmodia, with dimensions of 5.84–6.86 mm in width and 7.84–9.25 mm in length, while a plasmodia thickness ranged from 6.46–7.55 mm. Meanwhile, *K*. *rosenbuschi* plasmodia exhibited more constrained variations in length (6–7 mm), width (5.5 mm), and thickness (6.5 mm). In contrast, the newly identified *Kudoa* sp. displayed much smaller plasmodia measuring 4–4.7 mm (4.3 ± 0.3 mm) in length and 4.5–7 mm (6 ± 1.1 mm) in width. These distinctive plasmodia dimensions characterize it as a novel *Kudoa* species.

Additionally, this new *Kudoa* sp. featured distinctive spores, measuring 8–9 μm (8.6 ± 0.4 μm) 6–7.5 μm (6.9 ± 0.5 μm) in width. These spores were enclosed within milky-colored exudates within the plasmodia. The novel species also displayed distinctive polar capsules, measuring 2–5 μm (3.5 ± 0.5 μm) in length and 2.5–4.5 μm (3 ± 0.45 μm) in width. These measurements of spores and polar capsule is a defining feature of the new *Kudoa* species.

Recent advances in taxonomic classification have greatly benefited from molecular techniques, particularly the utilization of the 18S rDNA gene as a highly conserved genetic marker for distinguishing various myxosporean species. Nevertheless, certain *Kudoa* spp. exhibit minimal genetic variations in their 18S rDNA sequences when compared to closely related counterparts **[33].** For example, the genetic variance between *K*. *rosenbuschi* and *K*. *alliaria* is only two bp **[34].** Similar distinctions of two bp are observed between *K*. *chaetodoni* and *K*. *yasunagai* **[35]**, as well as between *K*. *hexapunctata* and *K*. *neothunni* **[33].**

In this study, the comprehensive genetic analysis aimed to distinguish the newly discovered *Kudoa* sp. from closely related species, especially *K*. *rosenbuschi* and *K*. *alliaria*, by analyzing the 18S rRNA gene. The results demonstrated a genetic divergence of about 1.1–1.17% within the 18S rRNA gene between the novel *Kudoa* sp. and its close counterparts. This level of divergence exceeds the commonly accepted threshold of 1.0% used in multiple studies for distinguishing between species based on genetic differences **[36–39].**

The recently identified *Kudoa* sp. exhibited marked deviations in plasmodia measurements, including length, width, thickness, and spore dimensions, setting it apart from other *Kudoa* genus members. Notably, when compared to closely related species like *K*. *rosenbuschi* and *K*. *alliaria*, which shared close genetic sequencing but occupied different geographical regions, our *Kudoa* sp. demonstrated noteworthy distinctions. Furthermore, even though it shared a common geographical range with the *Kudoa* spp. documented in Table 1, our newly identified *Kudoa* sp. displayed substantial differences in various aspects, including plasmodia dimensions and spore characteristics. Molecular analyses further validate these noteworthy distinctions, affirming the recent identification of a novel *Kudoa* sp. within the studied fish.

**Table 1:** *Kudoa* species identified in different fish species.

Species	Host (Site of infection)	Locality	Plasmodia (W/L) mm	Myxospores (w/L) um	Reference
*K*. *saudiensis*	*Rastrelliger kanagurta (*oocytes*)*	Red sea (Saudi Arabia)	4.3–5.4/2.4–3.6	1.2–1.8/1.1–1.4	[13]
*K*. *azevedoi*	*Trachurus trachurus (*Ovary*)*	Mediterranean Sea (Tunisia)	4.0–5.2/3.0–4.2	1.5–2.0/0.5–1.0	[7]
*K*. *crumena*	*Scomberomorus maculatus (*Skeletal muscles*)*	Atlantic Ocean, South Florida	9.3–10.4/6.8–8.2	3.2–4.6/2.1–2.9	[40]
*K*. *nova*	*Thunnus obesus*, *Trachurus sp*., *Neogobius sp*.,*Gobius sp*., *(*Skeletal muscles*)*	Atlantic Ocean, Black Sea, Mediterranean Sea	5.1–7.7/5.1–7.7	1.3–2.6	[32]
*K*. *quraishii*	*Rastrelliger kanagurta (*Skeletal Muscles*)*	Red Sea, Arabian Gulf (Saudi Arabia)	5.9–6.3/4.1–4.4	1.9–2.3/1.1–1.5	[15]
*K*. *scomberi*	*Scomber japonicus (*Muscles*)*	Japan	8.2–10.5/6.1–6.8	2.5–3.4/1.3–2.0	[41]
*K*. *pagrusi*	*Pagrus pagrus (*Heart*)*	Red Sea	5.8–7.2/6.5–8.6	2.6–4.2/1.0–1.8	[42]
*K*. *alliaria*	*Micromesistius australis Patagonotothen ramsay (*Muscles*)*	Argentina	5.84–6.86/7.84–9.25	5.84–6.86/7.84 - 9.25	[34]
*K*. *rosenbuschi*	*Merluccius gayii* *Merluccius hubbsi*	Argentina	6–7/5.5	6-7/5.5	[34]
*Kudoa sp*.	*Priacanthus tayenus* (Muscles)	Saudi Arabia	4–4.7/4.5–7	2–5 x 2.5–4.5	The present study

Histopathology revealed small white pseudocysts within muscle fibers, leading to adjacent muscle atrophy without inflammation, which aligns with prior observations **[10,43].** In contrast, infected Atlantic salmon showed pronounced inflammation when mature pseudocysts ruptured, releasing spores into muscle interstitial spaces, as noted by **[32].** These distinct host responses indicate that inflammatory effects of *Kudoa* muscle infections depend on pseudocyst integrity and maturation stage. Intact pseudocysts induce minimal inflammation, while ruptured pseudocysts triggering spore release cause pronounced reactions. Postmortem fish softening is due to proteolytic enzymes from *Kudoa* species. However, the source of these enzymes at different parasite maturation stages remains unidentified. Moribund fish with *Kudoa* spp. quickly develop a gelatinous substance even when refrigerated **[44–46]** Careful examination of samples with "soft flesh" symptoms is crucial to prevent misidentifying them as spoiled due to storage or transportation issues. This underscores the need for a thorough muscle inspection to identify spores. Consequently, *Kudoa* pseudocysts lead to the commercial rejection of infected fish due to postmortem myoliquefaction via myxospore release **[4,30].**

### Conclusion

This study identifies a novel *Kudoa* sp. in the skeletal musculature of the economically valuable purple-spotted bigeye, *P*. *tayenus* at the Jubail landing site in Saudi Arabia Gulf. Comprehensive examination, including morphology, molecular techniques, and histopathology, firmly establishes this parasite as a distinct taxonomic entity due to its unique plasmodial size, spore morphology, and genetic differences. This discovery expands the biodiversity of the threatened Arabian Gulf, broadening the known host range and geographic distribution of *Kudoa* parasites. The presence of this new species in a commercially fished population emphasizes the need for research on its life cycle and transmission dynamics to develop targeted management strategies. Our study also reveals the potential of this parasite to cause muscular atrophy and degradation in infected fish, highlighting the importance of assessing its impact on seafood quality and safety.
